# The impact of the five-factor model of personality on the performance of basketball players

**DOI:** 10.3389/fspor.2026.1766462

**Published:** 2026-03-04

**Authors:** Islam Mohammad Abbas

**Affiliations:** Department of Sport Science, Faculty of Sport Science, Arab American University, Jenin, Palestine

**Keywords:** athlete development, basketball performance, big five, personality traits, sports psychology, team sports

## Abstract

This research examined how the Five-Factor Model (Openness to Experience, Conscientiousness, Extraversion, Agreeableness, and Neuroticism) acts upon professional basketball players, exploring in addition whether these personality traits differ among playing position or levels of experience. A sample frame of 116 male professional players participating in the FIBA Asia Cup 2025 qualification was utilized. Personality traits were assessed using the 41-item Goldberg inventory, while performance was determined through a customized Player Efficiency index based on multiple linear regression analysis of core game statistics (Points, Rebounds, Assists, and Steals). The results indicated statistically Extraversion (*α* ≤ 0.05); no significant association was found for Agreeableness and Neuroticism. Even though significant differences were found by playing position for Agreeableness, only guards scored less than forwards and centers. Across levels of experience, there were significant differences for Openness to Experience, Conscientiousness, Extraversion, and Neuroticism. Players with more than ten years of experience had higher Openness and Conscientiousness and were lower on Neuroticism than players with five years of experience or less. Overall, the findings suggest that important personality traits—especially openness, self-discipline, and social engagement—can contribute powerfully to basketball performance. These results demonstrate the potential for integrating personality assessment into talent identification, player development programs, and team-building processes in elite basketball settings.

## Introduction

Personality has long been recognized as a fundamental psychological factor that influences human behavior and decision-making. It also affects performance across various areas of life, including competitive sport. From a trait perspective, personality is conceptualized as the relatively stable differences between individuals that predispose them to think, feel and behave consistently in different situations (1). In elite sport contexts, where physical abilities are often relatively comparable, such dispositional characteristics may play a particularly important role in explaining performance-related differences. This view is significant positive correlations between performance and Openness to Experience, Conscientiousness, and consistent with sport psychology frameworks emphasizing personality as a key determinant of sport behavior, motivation, and performance adaptation (2). In such settings, dispositional characteristics may partly explain individual differences in performance and career longevity. The Five-Factor Model (FFM), comprising Openness to Experience, Conscientiousness, Extraversion, Agreeableness, and Neuroticism, provides a robust and empirically supported framework for examining personality in the context of sport psychology. Previous research has shown that certain traits are modestly but consistently associated with athletic performance. These traits include conscientiousness and extraversion, particularly in team sports (3, 4). However, findings remain heterogeneous, suggesting that the relationships between personality and performance are contingent on contextual factors such as sport type, playing role, and competitive experience.

Contemporary theoretical models emphasize that personality does not influence performance in a purely direct manner. Instead, personality traits may exert indirect effects through motivation, training adherence, coping strategies, communication patterns, and team processes. Longitudinal evidence supports this dynamic perspective, suggesting that the relationship between personality traits and physical activity is bidirectional rather than static. For example, conscientiousness and openness have been shown to predict subsequent increases in physical activity, while engagement in physical activity may, in turn, foster increases in openness and conscientiousness over time (5). Meta-analytic findings further indicate that stable personality traits are systematically related to patterns of physical activity and inactivity across adulthood (6). Although much of this evidence originates from general physical activity contexts, these processes are theoretically relevant to elite sport environments, where prolonged exposure to structured training, competitive demands, and performance pressure may both select for and reinforce specific personality characteristics. Accordingly, these findings highlight the importance of considering developmental and contextual moderators—such as age and accumulated sport experience—when examining personality–performance relationships. Moreover, the bidirectional personality–context hypothesis proposes a reciprocal relationship, whereby personality shapes sport participation and performance, while prolonged engagement in elite sport may also select for—or further develop—certain personality characteristics. Empirical evidence from competitive football further supports this perspective. Research has shown that more experienced and adult athletes tend to score higher on Openness to Experience and Conscientiousness and lower on Neuroticism, with Openness and Conscientiousness positively associated with performance and Neuroticism negatively related to performance outcomes (7). These findings support both the selection and development hypotheses, suggesting that personality traits can both influence competitive sport performance and evolve through sustained sport participation. Complementary evidence based on the Millon model indicates that performance consistency among elite football players is linked to underlying motivational and cognitive personality characteristics, reinforcing the view that personality–performance relationships are multifactorial and context-dependent (8).

In basketball, performance demands vary substantially by playing position, requiring different cognitive, emotional, and interpersonal competencies. Despite this, empirical research examining how Five-Factor Model–personality traits relate to performance across basketball positions and experience levels remains limited. Most existing studies focus on general athlete populations or other sport disciplines, leaving a notable gap in elite basketball contexts.

Addressing this gap, the present study investigates the relationship between the Five-Factor Model personality traits and objective performance indicators among professional male basketball players competing at the international level. Additionally, it examines whether personality traits differ according to playing position and competitive experience, thereby contributing to a more nuanced understanding of how personality operates within high-performance team sport environments.

The performance hypothesis proposes that specific personality traits, most notably, conscientiousness and extraversion, are positively associated with athletic performance, whereas traits such as neuroticism may show weaker or negative associations, depending on sport type and context (4). These associations are largely shaped by contextual and psychological mediators, including motivational orientations, stress regulation, and interpersonal functioning within team settings (3).

Further investigation into the association between basketball performance and the Five-Factor Model (FFM) of personality could significantly contribute to advancing knowledge in key domains such as talent identification, psychological conditioning, and athlete development in competitive sports. A deeper comprehension of the role personality traits play in athletic performance may offer critical insights for optimizing player development frameworks, enhancing team cohesion, and customizing coaching methodologies to align with individual psychological profiles. By recognizing and fostering personality traits linked to superior on-court performance, coaches, sports psychologists, and team managers can better support individual athletic development and overall team functioning.

### Literature review

#### Conscientiousness and extraversion

Conscientiousness and extraversion have frequently been identified as important predictors of athletic success. Extraversion, in particular, has been consistently linked to success in team sports and is characterized by sociability, assertiveness, and high energy levels. Athletes with higher levels of extraversion may excel in team sports such as volleyball, basketball, and soccer. In these sports, leadership, communication, and social interaction are essential for team cohesion and success (9). High extraversion is beneficial in team sports and endurance events, where social interaction and energy are critical (10). *A meta-analysis by Yang et al. reported positive correlations between these traits and athletic performance, indicating that athletes who exhibit higher levels of sociability and diligence tend to achieve superior performance outcomes.* This relationship is particularly pronounced in basketball, where teamwork and consistent practice are critical (11). Conscientious athletes, characterized by reliability, motivation, and organizational skills, demonstrate a heightened capacity for success in such collaborative and structured environments. *Conversely, athletes with more introverted tendencies—characterized by introspection and a preference for solitary activities—may demonstrate greater proficiency in individual sports such as golf, swimming, or tennis.* where sustained focus and intrinsic motivation are essential. Empirical evidence suggests that athletes generally exhibit higher levels of extraversion compared to non-athletes. Team sport participants also display significantly greater extraversion than those engaged in individual sports. These findings highlight the potential influence of personality traits on sport selection and performance, emphasizing the role of extraversion in team-based athletic environments (12). A review by Piedmont et al. demonstrated that higher levels of conscientiousness were associated with better coachability, stronger work ethic, and enhanced athletic ability (13). High levels of conscientiousness are critical for athletic success, as this trait encompasses endurance, discipline, and goal-oriented behavior. These attributes enable athletes to engage in strategic planning, maintain sustained focus, and effectively navigate challenges, thereby enhancing their overall performance and resilience in competitive environments (10). Extraversion and conscientiousness were significantly related to sports performance at all levels (11, 14).

#### Openness to experience and agreeableness

Openness to Experience reflects cognitive flexibility, curiosity, creativity, and a willingness to engage with novel ideas and strategies. Within sports contexts, openness has been proposed as a trait that may facilitate adaptability, learning, and tactical innovation. Although some studies have reported positive associations between openness and athletic performance, findings remain inconsistent across sports and competitive levels (4, 14). In basketball, openness may facilitate adaptability and strategic creativity, while agreeableness can enhance team cohesion and collaborative play. However, the precise influence of these traits on basketball performance requires further investigation. Zar et al. reported that significant correlations were identified between agreeableness and athletic performance at the provincial (r = 0.133, *p* = 0.010) and national levels (r = 0.171, *p* = 0.001). However, no statistically significant relationship was observed at the international level, suggesting that the influence of agreeableness on performance may vary depending on the competitive context. In specific contexts, neuroticism may detrimentally affect performance, while agreeableness can confer distinct advantages (11, 14).

#### Neuroticism

Neuroticism, which reflects emotional instability and heightened stress reactivity, has frequently been linked to poorer athletic outcomes. Athletes with high levels of neuroticism may experience increased anxiety and stress, negatively affecting their performance. In team sports such as basketball, where emotional control under pressure is essential, elevated neuroticism may undermine on-court effectiveness. Król-Zielińska et al. reported that low neuroticism indicates emotional stability, which helps athletes manage stress and perform consistently. This outcome is important, as emotional resilience enables athletes to maintain focus under high-pressure conditions. Additionally, it reduces the likelihood of mental health challenges, such as burnout or anxiety, while enhancing an athlete's ability to recover effectively from setbacks or injuries (10). These factors collectively contribute to sustained performance and long-term well-being in competitive sports. Athletes with lower levels of neuroticism (i.e., greater emotional stability) tend to receive higher performance ratings from coaches (13). Neuroticism demonstrated a significant negative correlation with sports performance across all competitive levels, indicating that athletes with lower levels of neuroticism tend to exhibit greater emotional stability, resilience, and enhanced stress management capabilities. These attributes contribute to their ability to perform consistently and effectively in high-pressure sporting environments (14). In summary, previous research highlights the relevance of personality traits—particularly conscientiousness and extraversion—in athletic performance, while also emphasizing the moderating role of sport type, competitive level, and performance measurement methods. Despite this growing literature, limited attention has been given to position-specific and experience-related personality differences in elite basketball, underscoring the need for the present investigation, particularly focusing on the Five-Factor Model. These studies suggest that conscientiousness and extraversion are positively correlated with athletic performance, indicating that athletes who are more disciplined, goal-oriented, and sociable tend to perform better. Neuroticism generally shows a negative correlation, meaning that higher levels of anxiety and emotional instability can hinder performance. The findings also emphasize the importance of moderating factors such as sport type, gender, publication type, and performance measurement methods, which contribute to the heterogeneity observed across studies. For instance, team sports might benefit more from extraversion due to the social dynamics involved, whereas individual sports may rely more heavily on conscientiousness. Additionally, the differences in results between perceived and actual performance measurements highlight the complexity of assessing athletic success purely through personality metrics. Overall, these insights provide a foundation for understanding how psychological attributes can influence athletic outcomes and offer practical applications for coaching and athlete development strategies. Despite the recognition of personality's role in sports psychology, there is a lack of research investigating the specific relationship between FFM personality traits and basketball positions (i.e., center, forward, guard). The current literature primarily focuses on the general psychological attributes of successful athletes, such as mental toughness, motivation, and stress management (3). However, little is known about how the unique demands of different basketball positions align with distinct personality traits. For example, do extraverted players perform better as guards due to their leadership and communication skills? Are highly conscientious players more effective as centers because of their defensive discipline? Do highly neurotic players struggle under high-pressure game situations? Addressing these questions is essential for advancing talent identification, psychological training, and strategic team formation in basketball. This study aims to investigate the influence of the Five-Factor Model (FFM) personality traits—Neuroticism, Extraversion, Openness, Agreeableness, and Conscientiousness—on the performance of basketball players, with a focus on identifying specific personality characteristics that contribute to enhanced individual and team performance in competitive settings.

### Hypotheses

This study aims to investigate

- There is no statistically significant relationship at the significance level (*α* ≤ 0.05) between the Five-Factor Model personality traits (openness to experience, conscientiousness, extraversion, agreeableness, and neuroticism) and the performance of basketball players.
- There are no statistically significant differences, at the significance level (*α* ≤ 0.05) in the Five-Factor Model personality traits (openness to experience, conscientiousness, extraversion, agreeableness, and neuroticism) among basketball players according to their playing positions (guards, forwards, centers).
- There are statistically significant differences, at the significance level (*α* ≤ 0.05), in the Five-Factor Model personality traits among basketball players according to their levels of playing experience.

### Research methodology

#### Participants

total of one hundred sixteen (116) male professional basketball players (mean age 30.71 ± 4.56; age range = 22–35 years), all of whom were actively participating in the FIBA Asia Cup 2025 qualification, were recruited using a purposive (criterion-based) sampling approach. voluntarily participated in this study. The players represented different national teams. Prior to data collection, all participants were informed about the objectives of the study. They were also informed of their right to withdraw at any stage without providing any justification by choosing not to complete the questionnaire. The study protocol received ethical approval from the Committee on Ethics of Scientific Research at the Arab American University, under the reference number J-2025/A/3/N, ensuring compliance with recognized ethical standards for research involving human participants, The study sample consisted of 37 guards (31.9%), 46 forwards (39.7%), and 33 centers (28.4%). Regarding playing experience, 35 players (30.2%) had ≤5 years of professional experience, 45 players (38.8%) had 6–10 years, and 36 players (31.0%) had more than 10 years of experience (see Table 1)

**Table 1:** Distribution of study sample according to playing position & experience Variable (*n* = 116).

Variable	Position Levels	Frequency	Percentage
Playing Position	Point Guard	37	31.9%
	Forward	46	39.7%
	Center	33	28.4
	Total	**116**	**100%**
Playing Experience	5 ≥	35	30.2%
	6–10 years	45	38.8%
	> 10 years	36	31%
	Total	**116**	**100%**

#### Assessment procedure

Data collection was conducted using an online questionnaire prior to the start of the FIBA Asia Cup 2025 qualification tournament. Given the elite nature of the sample, participants were recruited through direct coordination with team administrators, who acted as gatekeepers and distributed the electronic survey link to eligible players. The researcher, a former Jordanian national team basketball player, used existing professional networks to facilitate access to team administrators. Players completed the questionnaire individually at their convenience before the competition period. Participation was voluntary, and confidentiality and anonymity were assured. No identifying information was collected, and the researcher had no involvement in players' responses.

### Study tool

#### Performance indices

Game statistics for each player were collected from the most recent matches during the 1st Round (6 Games) of qualifications for the FIBA Asia Cup 2025. These statistics included points (the total number of points scored), assists (the number of times a player facilitated a teammate's score), rebounds (the number of times a player retrieved the ball after a missed shot), steals (the number of times a player intercepted the ball from the opponent), games played (the total number of games participated in by each player). These metrics were calculated for each player across the qualifications for the FIBA Asia Cup 2025a comprehensive indicator of their athletic performance. Position-specific averages revealed that guards averaged 9.0 points, 2.1 assists, and 0.6 steals per game, and 3.0 rebounds per game with averaged 4.13 game played; forwards averaged 8.8 points, 1.8 assists, and 1.0 steals per game and 3.5 rebounds per game with averaged 4.47 games play; and centers averaged 6.2 points, 0.9 assists, and 0.7 steals per game and 4.7 rebounds per game with averaged 4.46 games play. These measures provide a detailed assessment of each player's contribution and effectiveness within their respective roles on the team.

In order to design a customized formula for evaluating individual player performance in basketball, statistical analysis was conducted using Multiple Linear Regression. The general efficiency index (Efficiency Index) was used as the dependent variable, while Points, Rebounds, Assists, and Steals were treated as independent variables. Following the analysis in (SPSS, Version 26), the study relied on the Standardized Beta Coefficients to determine the exact relative weights of each variable. These coefficients were chosen as they allow for a direct comparison of the relative importance of each predictor, regardless of differences in their original measurement units. Beta coefficients reflect the proportional contribution of each variable to overall efficiency after adjusting for scale differences among variables. The regression results indicated that the most influential variable in explaining player efficiency was Points Scored (PTS), followed by Rebounds (REB), then Assists (AST), and finally Steals (STL). These results were used to derive relative weights incorporated into the proposed evaluation formula, as illustrated in the following (Table 2):

**Table 2:** Standardized Beta coefficients and assigned relative weights for player performance variable*s.*

Variable	Standardized beta	Percentage-based relative weight
Points (PTS)	0.593	53.4%
Rebounds (REB)	0.275	24.8%
Assists (AST)	0.177	15.9%
Steals (STL)	0.066	5.9%

All independent variables are statistically significant (Sig. < 0.05) with no indication of multicollinearity (VIF < 5), confirming the suitability of the regression model.

Based on these weights, the following formula was constructed to calculate the relative player efficiency: Player_Efficiency=(0.534 × PTS) + (0.248 × REB) + (0.159 × AST) + (0.059 × STL).

This formula represents a quantitative and scientifically valid expression of the relative significance of each statistical indicator in performance evaluation, providing an objective method for comparing player efficiencies based on the available game statistics.

### Personality questionnaire

41-item inventory that measures an individual on the Five-Factor Model (dimensions) of personality (Goldberg, 1993). Each of the factors is then further divided into personality facets. The Big Five Factors are chart recreated from John & Srivastava (15). The Goldberg inventory was selected due to its strong psychometric properties, widespread use in personality research, and practical suitability for applied sport settings. Compared with more extensive instruments such as the NEO-FFI, this measure provides a concise and time-efficient assessment, which was particularly appropriate given the elite nature of the sample and the data collection context prior to an international competition. Moreover, the focus of the present study was on the assessment of broad personality dimensions rather than facet-level distinctions, making the Goldberg inventory an appropriate and methodologically sound choice. In order to study the basic personality traits, The inventory is based on one of the most popular concepts, engaging personality in terms of five features called dimensions or factors (FFM): Neuroticism, Extraversion, Openness To Experience, Agreeableness, Conscientiousness Each item is a statement demanding answers from the teste on a five-point scale from 1 to 5, where 1 is the answer - “strongly disagree” and 5 is - “strongly agree”. And To ensure the reliability of the instrument, the Cronbach's Alpha coefficient was calculated for each of the five dimensions. All coefficients indicated acceptable levels of internal consistency, confirming the reliability of the scale in measuring the targeted personality constructs within the sample.

The results presented in (Table 3) indicate that all five personality dimensions demonstrated acceptable to high levels of internal consistency, as reflected by their respective Cronbach's Alpha coefficients. Specifically:

- Openness to Experience (*α* = 0.814), Conscientiousness (*α* = 0.926), Extraversion (*α* = 0.771), and Agreeableness (*α* = 0.799) exhibited good to excellent reliability.
- Neuroticism showed acceptable reliability with a Cronbach's Alpha of 0.735.
- The overall scale reliability reached 0.909, indicating a high level of internal consistency for the total instrument.

These findings confirm the adequacy and reliability of the measurement tool for assessing the Five-Factor Model personality traits in the study sample

**Table 3:** Reliability coefficients (cronbach's alpha) for the five-factor model personality traits.

Personality Traits	Cronbach's Alpha	N of Items
Openness to experience	0.814	8
conscientiousness	0.926	9
Extraversion	0.771	7
Agreeableness	0.799	8
Neuroticism	0.735	9
Total Scale Reliability	0.909	41

### Study variables

Independent Variable: Five-Factor Model (dimensions) of personality.

- openness to experience
- conscientiousness
- Extraversion
- Agreeableness
- Neuroticism

Dependent Variables: Players Performance indices

### Statistical treatments

The researcher employed the Statistical Package for the Social Sciences (SPSS) software to perform the following analyses:

- Frequencies and percentages were calculated, to describe the distribution of participants according to their playing positions (Point Guard, Forward, Center) and playing experience categories (≤5 years, 6–10 years, >10 years).
- One-Way ANOVA to identify differences between basketball players across the three playing position groups in relation to their Five-Factor Model personality traits and performance indices.
- Pearson's correlation analysis was conducted to investigate the relationships between the Five-Factor Model personality traits and basketball players’ performance.
- To control for Type I error associated with multiple comparisons, Tukey's Honestly Significant Difference (HSD) test was applied for *post hoc* analyses following significant one-way ANOVAs.

## Result

### First

There is no statistically significant relationship at the significance level (*α* ≤ 0.05) between the Five-Factor Model personality traits (openness to experience, conscientiousness, extraversion, agreeableness, and neuroticism) and the performance of basketball players.

To answer this hypothesis the researcher applied Pearson's Simple Correlation Coefficient to examine the significance of the relationship between the Five-Factor Model personality traits (openness to experience, conscientiousness, extraversion, agreeableness, and neuroticism) and the performance of basketball players. (Table 4) presents the relevant findings.

**Table 4:** Results of Pearson's correlation test between the five-factor model personality traits and players performance.

Personality Trait	r	*p*-value	Statistical significance
Openness to Experience	0.266	0.004*	Significant
Conscientiousness	0.228	0.001*	Significant
Extraversion	0.187	0.045*	Significant
Agreeableness	0.035-	0.705	Not Significant
Neuroticism	0.028	0.763	Not Significant

* *^α^* ^≤^ ^0.05.^

Results shown in Table 4 indicate a weak yet valid positive correlation between the trait Openness to Experience and performance in basketball players, having a Pearson correlation coefficient of (r = 0.266) at a significant value of (*p* = 0.004), which is below the accepted threshold of (*α* ≤ 0.05). This implies that players with high openness may adapt more readily to new strategies and think creatively. They may also react flexibly to dynamic in-game conditions, which could enhance their performance. Conscientiousness has been found to be another personality trait that has significantly but positively related to performance (r = 0.228, *p* = 0.001). What this suggests, therefore, would be that those players who are very organized, responsible and self-disciplined may improve their chances of maintaining similar performance levels over time, and may meet the demands of competition. Extraversion, on the other hand, was shown to correlate weakly yet significantly positively with performance (r = 0.187, *p* = 0.045). This means that a more outgoing and socially energetic player will benefit from enhanced communication, motivation, and team dynamics, which will all contrive to the better performance. Otherwise, the other aspects of personality, Agreeableness (r = −0.035, *p* = 0.705) and Neuroticism (r = 0.028, *p* = 0.763), were not statistically correlated with the performance, indicating no observable association between these traits and objective performance indices in the present sample. These outcomes indicate that characteristics such as interpersonal harmony and emotional instability do not seem to affect performance in this sample of basketball players.

### Second

There are no statistically significant differences, at the significance level (*α* ≤ 0.05) in the Five-Factor Model personality traits (openness to experience, conscientiousness, extraversion, agreeableness, and neuroticism) among basketball players according to their playing positions (guards, forwards, centers).

To answer this hypothesis, the researcher conducted a One-Way ANOVA to examine significant differences in the Five-Factor Model personality traits among basketball players based on their playing positions. Tables 5, 6 presents the results of the analysis while (Table 7) shows the results of *post hoc* tests.

**Table 5:** Means and standard deviations of the five-factor model personality traits among basketball players in palestine based on playing positions.

Playing Position personality traits	Point Guard		Forward		Center	
	Mean	Standard Deviation	Mean	Standard Deviation	Mean	Standard Deviation
Openness to Experience	3.40	0.835	3.51	0.806	3.53	0.751
Conscientiousness	3.28	1.192	3.52	0.952	3.45	0.897
Extraversion	3.31	0.704	3.57	0.830	3.48	0.912
Agreeableness	3.37	0.610	3.72	0.388	3.80	0.587
Neuroticism	3.32	0.556	3.49	0.645	3.29	0.652

**Table 6:** Results of One-Way ANOVA for differences in five-factor model personality traits Among basketball players by playing position.

Variables	Source of Variation	Sum of Squares	Degrees of Freedom (df)	Mean Square	F-value	Significance Level (p)	Statistical Significance	*η* ^2^
Openness to Experience	Between Groups Within Groups Total	0.332 72.508 72.841	2 113 115	0.166 0.642	0.259	0.772	Not Significant	0.004
Conscientiousness	Between Groups Within Groups Total	1.256 117.812 119.068	2 113 115	0.628 1.043	0.602	0.549	Not Significant	0.010
Extraversion	Between Groups Within Groups Total	1.410 75.593 77.004	2 113 115	0.705 0.669	1.054	0.352	Not Significant	0.018
Agreeableness	Between Groups Within Groups Total	3.831 31.241 35.072	2 113 115	1.915 0.276	6.928	0.001*	Significant	0.109
Neuroticism	Between Groups Within Groups Total	0.922 43.510 44.431	2 113 115	0.461 0.385	1.197	0.306	Not Significant	0.020

* Significance Level: (*α* ≤ 0.05).

**Table 7:** Tukey HSD *post hoc* comparisons for agreeableness scores by playing position.

Comparison	Mean Difference	*p*-value	Statistical Significance
Guard vs. Forward	0.348-	0.009*	Significant
Guard vs. Center	0.431-	0.002*	Significant
Forward vs. Center	0.082	0.769	Not Significant

* Significance Level: (*α* ≤ 0.05).

Descriptive statistics indicated noticeable differences in personality trait profiles across playing positions. Guards exhibited lower mean scores on Agreeableness (M = 3.37) compared to forwards (M = 3.72) and centers (M = 3.80). Forwards and centers showed slightly higher mean levels of Openness to Experience (M = 3.51 and M = 3.53, respectively) compared to guards (M = 3.40). Regarding Conscientiousness and Extraversion, forwards demonstrated the highest mean scores (M = 3.52 and M = 3.57, respectively), followed by centers and guards. Mean levels of Neuroticism were relatively comparable across positions, with minor variations observed between groups. Detailed descriptive statistics by playing position are presented in Table 5.

A one-way analysis of variance (ANOVA) revealed a statistically significant effect of playing position on Agreeableness (F = 6.93, *p* = 0.001), with an eta squared value of *η*^2^ = 0.109, indicating a moderate effect size. This suggests that playing position accounted for approximately 10.9% of the variance in agreeableness scores. *post hoc* comparisons demonstrated that guards scored significantly lower on agreeableness than forwards (Mean Difference = −0.348, *p* = 0.009) and centers (Mean Difference = −0.431, *p* = 0.002). In contrast, no statistically significant difference was observed between forwards and centers (Mean Difference = 0.082, *p* = 0.769). The results of the ANOVA and *post hoc* analyses are presented in Tables 6, 7.

### Third

There are statistically significant differences, at the significance level (*α* ≤ 0.05), in the Five-Factor Model personality traits among basketball players according to their levels of playing experience.

To answer this hypothesis, the researcher conducted a One-Way ANOVA to examine significant differences in the Five-Factor Model personality traits among basketball players based on their playing Experience. Tables 7, 8 presents the results of the analysis while (Table 9) shows the results of *post hoc* tests.

**Table 8:** Means and standard deviations of the five-factor model personality traits Among basketball players in palestine based on playing experience.

Playing Experience personality traits	5 ≥		6–10 years		• > 10 years	
	Mean	Standard Deviation	Mean	Standard Deviation	Mean	Standard Deviation
Openness to Experience	3.49	0.921	3.17	0.855	3.86	0.256
Conscientiousness	3.40	1.023	3.10	1.272	3.85	0.165
Extraversion	3.67	0.861	3.17	0.949	3.63	0.404
Agreeableness	3.76	0.635	3.50	0.607	3.67	0.323
Neuroticism	3.52	0.722	3.44	0.615	3.18	0.469

**Table 9:** Results of One-Way ANOVA for differences in five-factor model personality traits Among basketball players by playing experience.

Variables	Source of Variation	Sum of Squares	Degrees of Freedom (df)	Mean Square	F-value	Significance Level (p)	Statistical Significance	η^2^
Openness to Experience	Between Groups Within Groups Total	9.496 63.344 72.841	2 113 115	4.748 0.561	8.470	0.001*	Significant	0.130
Conscientiousness	Between Groups Within Groups Total	11.212 107.856 119.068	2 113 115	5.606 0.954	5.873	0.004*	Significant	0.094
Extraversion	Between Groups Within Groups Total	6.367 70.637 77.004	2 113 115	3.183 0.625	5.092	0.008*	Significant	0.082
Agreeableness	Between Groups Within Groups Total	1.449 33.623 35.072	2 113 115	0.724 0.298	2.435	0.092	Not Significant	0.041
Neuroticism	Between Groups Within Groups Total	2.302 42.129 44.431	2 113 115	1.151 0.373	3.088	0.049*	Significant	0.051

* Significance Level: (*α* ≤ 0.05).

Descriptive analyses by playing experience revealed systematic variations in personality trait levels across experience groups. Players with more than 10 years of experience demonstrated the highest mean scores for Openness to Experience (M = 3.86) and Conscientiousness (M = 3.85), whereas players with 5 years of experience or less showed comparatively lower mean values on these traits (M = 3.49 and M = 3.40, respectively).In contrast, Neuroticism tended to be lower among players with more than 10 years of experience (M = 3.18) compared to less experienced players. Mean levels of Agreeableness and Extraversion showed smaller differences across experience groups. Full descriptive statistics by experience level are reported in Table 8.

Post hoc comparison results revealed statistically significant differences in several Five-Factor Model personality traits across levels of playing experience. For Openness to Experience, players with more than 10 years of experience scored significantly higher than those with 5 years of experience or less (Mean Difference = −0.368, *p* = 0.041) and those with 6–10 years of experience (Mean Difference = 0.688, *p* = 0.001). The corresponding one-way ANOVA indicated a significant effect of playing experience on openness (F = 8.47, *p* = 0.001), with an eta squared value of *η*^2^ = 0.130, reflecting a moderate effect size. For Conscientiousness, *post hoc* analyses showed that players in the 6–10 years' experience group scored significantly lower than those with more than 10 years of experience (Mean Difference = −0.748, *p* = 0.001). The ANOVA results demonstrated a significant effect of playing experience on conscientiousness (F = 5.87, *p* = 0.004), with *η*^2^ = 0.094, indicating a moderate effect size. Significant *post hoc* differences were also observed for Extraversion, with the 6–10 years’ experience group scoring significantly lower than both the ≤5 years group (Mean Difference = −0.502, *p* = 0.006) and the >10 years group (Mean Difference = −0.456, *p* = 0.011). The corresponding ANOVA yielded a significant effect (F = 5.09, *p* = 0.008), with an eta squared value of *η*^2^ = 0.082, representing a small-to-moderate effect size. For Neuroticism, a statistically significant *post hoc* difference was identified only between players with ≤5 years of experience and those with more than 10 years of experience (Mean Difference = 0.341, *p* = 0.020). The ANOVA indicated a significant effect of playing experience on neuroticism (F = 3.09, *p* = 0.049), with *η*^2^ = 0.051, corresponding to a small effect size. No statistically significant *post hoc* differences were observed for Agreeableness, consistent with the non-significant ANOVA result (F = 2.44, *p* = 0.092, *η*^2^ = 0.041). The results of the ANOVA and *post hoc* analyses by playing experience are presented in Tables 9, 10.

**Table 10:** Tukey HSD *post hoc* comparisons for personality traits across playing experience.

Personality Traits	Comparison	Mean Difference	*p*-value	Statistical Significance
Openness to Experience	5 ≥ vs. **>** 10 years	0.368-	0.041*	Significant
	6–10 years vs. **>** 10 years	0.688	0.001*	Significant
Conscientiousness	6–10 years vs. **>** 10 years	0.748-	0.001*	Significant
Extraversion	5 ≥ vs. 6–10 years	0.502-	0.006*	Significant
	6–10 years vs. **>** 10 years	0.456-	0.011*	Significant
Neuroticism	5 ≥ vs. **>** 10 years	0.341	0.020*	Significant

* Significance Level: (*α* ≤ 0.05).

## Discussion

This study demonstrated a statistically significant positive association between Openness to Experience and performance among very experienced male basketball players (r = 0.266, *p* = 0.004). Although the magnitude of this correlation is low to modest, it still carries theoretical and practical relevance in performance psychology. These findings are consistent with previous research in team sports. For example, Ruiz-Barquín and García-Naveira reported that Openness to Experience and Conscientiousness were positively associated with sport performance, whereas Neuroticism showed a negative relationship, particularly among more experienced athletes. This convergence suggests that adaptive personality traits may facilitate performance across different team sport contexts, despite sport-specific demands (7).

Openness to Experience is a core dimension of personality. It reflects intellectual curiosity, cognitive flexibility, and receptivity to new ideas (4, 16). In elite sports, these traits are manifested by an athlete's willingness to pursue alternative training methods, innovative tactical strategies, and roles that may shift fluidly during the course of a game (17).

To contextualize these findings, it is important to consider how athletic performance was assessed in the present study. Performance measurement approaches may influence observed personality–performance associations. Athletic performance in the present study was assessed using objective performance indicators derived from official match statistics. Objective measures are widely regarded as more reliable and less susceptible to bias than subjective evaluations, such as self-reports or coach ratings, particularly in elite sport contexts. Previous research has shown that relationships between personality traits and performance may differ across competitive levels. More consistent associations are often observed among high-level athletes than among amateur players. This has been attributed to the reduced variability in physical skills and the increased importance of psychological factors in elite performance environments. Accordingly, the use of objective performance indices in a sample of professional basketball players competing at the international level provides an appropriate framework for examining personality–performance relationships. These traits are particularly important for experienced athletes. Such athletes possess extensive technical knowledge but must continuously adapt to fast-paced and tactically complex competitive environments. A study of basketball players found that the Five-Factor Model personality factors, including openness, can influence dynamic criteria such as effectiveness throughout a sports season (18).

Such behaviors may enhance tactical flexibility and performance capabilities.

This process may support sustainable performance over time through continuous learning and feedback integration (19). As athletes face the many challenges of their careers, openness to experience may act as a psychological buffer to facilitate coping, foster novelty, and build resilience. It is also important to note that while experience may enhance this quality by exposing athletes to different roles and demands, sometimes set routines may reduce openness as time goes on (17). Openness to Experience showed a statistically significant but small positive association with basketball performance. Although modest in magnitude, this finding is consistent with trait-based perspectives suggesting that cognitive flexibility may facilitate adaptation to dynamic performance demands in elite team sports. A further important nuance is that the magnitude of the openness–performance correlation observed in this study (r ≈.27) falls within the “typical” range for individual-differences research, where empirically derived benchmarks suggest that correlations of.10,.20, and.30 can be interpreted as small, medium, and large effects, respectively (20). This indicates that although openness is not a dominant determinant of performance, it is a non-trivial contributor that operates alongside physical, technical, and tactical factors to shape outcomes in elite basketball (11). In addition, the present results are consistent with recent multi-sport work showing that higher openness is linked to better adaptation to role changes, greater receptivity to coaching feedback, and more flexible coping with performance slumps, all of which are central to maintaining performance over a competitive season (4, 11, 18). In this sense, openness appears to function less as a “talent marker” and more as a psychological capacity that allows experienced players to translate their accumulated knowledge into context-sensitive decisions on the court, particularly when facing novel opponents or strategic innovations (4). From a practical standpoint, these findings imply that identifying players who score higher on openness may be beneficial. Such players may be more likely to experiment with new offensive sets, embrace analytically informed training adjustments, and assume unconventional roles (e.g., stretch centers, point forwards), thereby increasing tactical versatility at the team level (3, 4, 18). This interpretation is further supported by Díaz-Rodríguez and Pérez-Córdoba, who demonstrated that openness to experience is positively associated with creativity and flexibility in athletic decision-making, particularly among highly motivated athletes. These findings provide convergent evidence that openness enhances performance not by directly increasing physical output, but by facilitating adaptive, innovative, and context-sensitive decisions during competition (21).

Conscientiousness was positively associated with basketball performance among experienced players (r = 0.228, *p* = 0.001), although the magnitude of this relationship was small. According to this research, self-regulatory and goal-oriented tendencies may support more consistent performance outcome in elite sport settings. Self-discipline, organization, perseverance, and a strong sense of responsibility are common characteristics of conscientiousness, and these traits have been connected to effective training engagement and performance reliability (3, 22). These traits can be seen in basketball players who follow regimented training plans, make focused decisions during games, respond to coaching feedback, and consistently dedicate themselves to improving their skills. When taken as a whole, these behaviors may support teamwork and performance stability, which is in line with earlier studies that found conscientiousness to be a significant but non-dominant predictor of success and dependability in elite athletic settings (22, 23). Also, highly conscientious persons' attention to detail enables them to scrutinize their performances, understand their weaknesses, and employ remedial measures for improvement (4). The current findings thus affirm the conceptualization of conscientiousness as a primary trait for sustaining high performance in the athletic field, particularly in highly structured, sternly competitive environments such as professional basketball.

The present data also converge with meta-analytic evidence showing that conscientiousness is the most robust Five-Factor Model predictor of objective performance indices across sports, especially when performance is assessed via statistics and rankings rather than solely by self-report (11). In basketball, such reliability and task focus are likely to manifest in behaviors that do not always appear in traditional box-score metrics—such as consistently setting effective screens, maintaining correct spacing off the ball, and executing defensive rotations—which nevertheless accumulate into meaningful performance gains at the team level (22, 24). Moreover, the observed experience-related differences, whereby players with more than ten years of experience scored higher on conscientiousness than those with intermediate experience, suggest that conscientiousness may both predispose and be reinforced by sustained participation at higher competitive levels (18). Similar age- and experience-related patterns have been observed in competitive football, where adult athletes demonstrated higher levels of Openness to Experience and Conscientiousness and lower Neuroticism compared to younger players (7). These results support the notion that prolonged exposure to competitive sport may both select for and foster more adaptive personality profiles over time.

This aligns with longitudinal findings that athletes who remain in high-performance pathways tend to show increases in planning, persistence, and self-regulation over time, partly because those low in conscientiousness are more likely to drop out or plateau (4, 25). Consequently, integrating conscientiousness assessments into talent development systems could help identify players who are more likely to tolerate high training loads, adhere to individualized conditioning programs, and reliably implement complex tactical schemes, thereby reducing the risk of “wasted talent” due to poor self-management or inconsistent effort (4, 11, 23).

Thus, the present study established the existence of a significant positive correlation between extraversion and athletic performance in experienced basketball players (r = .187, *p* = .045). This modest effect, while consistent with earlier studies would indicate that extraversion may facilitate certain activities in team sports, where high interpersonal activity, communication, and responsiveness are demanded (3, 23). Extraversion has been primarily characterized as personality orientation which includes sociability, assertiveness, energy, and a preference for stimulating social environments (4, 26). In the context of competitive basketball, these types of characteristics may be displayed in terms of effective game-time interaction, emotional channeling, and proactive involvement with team dynamics. Extraverted athletes are more inclined to initiate involvement with teammates, request feedback, and seek active roles in high-stakes and socially challenging situations thereby leading to successful performance (16). Furthermore, highly-extraverted experienced players take on leadership responsibility, showing initiative in coordinating game plans while administering team spirit-building activities, both of which are vital to team cohesion and requisite to achieving success (26). Thus, the findings of this study further strengthen the understanding of extraversion as a personality construct beneficial in elite team sports. It enhances the motivation and emotional strength of individuals for individual performance while enhancing communication and coordination to benefit teams. This is an asset in dynamic sports such as basketball, where social flexibility and fast-paced interpersonal decision-making are critical. The strength and direction of this association mirror broader evidence that extraversion is modestly but consistently related to higher sport participation and, in many contexts, to superior performance, particularly in team and invasion games where communication, emotional expressiveness, and on-court presence are central (11, 12). In addition, positional demands may shape how extraversion is expressed and rewarded. Guards, who typically orchestrate offensive sets and serve as primary communicators with coaches, may benefit more from assertiveness and verbal expressiveness, whereas centers can leverage extraversion by vocally coordinating defensive coverages and box-out assignments in the paint (16, 27). The current findings, taken together with prior evidence that leadership roles in sport tend to be occupied by more extraverted athletes, support the view that extraversion contributes to performance largely through its influence on communication, perceived leadership, and the regulation of collective efficacy within the team (16, 26). Practically, this suggests that coaches might not only look for extraverted players to occupy playmaking and captaincy roles but also provide structured opportunities (e.g., pre-game briefings, in-game “huddles”) in which these athletes can use their social energy to coordinate teammates and maintain a high-intensity yet controlled emotional climate during competition (3, 12). These findings are further supported by Díaz-Rodríguez et al. who reported a positive association between extraversion and engagement in physical activity, particularly in group-based sports contexts. This behavioral tendency toward socially interactive sporting environments may partially explain why extraverted athletes demonstrate performance advantages in team sports such as basketball (28).

The results of the present study did not indicate any statistically significant relationship of Agreeableness (r = −0.035, *p* = 0.705) or Neuroticism (r = 0.028, *p* = 0.763) with sports performance in experienced basketball players. This shows that these two personality traits may not be strong indicators of performance in elite team sports, at least for this specific sample.

Agreeableness-or tendency toward trust, empathy, cooperation, and altruism-might have a limited direct bearing on the competitive and high-pressure environment of elite basketball, where assertion, rapid decision-making, and competitive drive often immediately affect performance (23). Although agreeableness can encourage positive interpersonal dynamics and help facilitate effective conflict resolution, it may be that there is a more indirect and contextual influence on measurable athletic performance. This notion is supported by Al-Yaaribi and Kavussanu, who emphasize that agreeableness, though not strongly tied to individual performance indices, plays a vital role in fostering team cohesion, mutual respect, and collective efficacy (29).

Similarly, the absence of a significant association between neuroticism and athletic performance aligns with previous literature suggesting that high-performance athletes often develop psychological coping mechanisms that attenuate the potential negative effects of neurotic tendencies, such as anxiety, emotional volatility, and stress reactivity. A review by Shuai et al. and Piepiora suggests that while high neuroticism can present challenges for basketball players, its impact on athletic performance is not deterministic (4, 16). In experienced players, prolonged exposure to competitive environments may buffer against the disruptive influence of neuroticism, thereby diminishing its predictive value for performance. The relationship between neuroticism and athletic performance is not isolated but rather interacts with other personality traits within the Five-Factor Model (4). For instance, high conscientiousness, characterized by discipline and responsibility, may help players with high neuroticism to structure their training and preparation, providing a sense of control and reducing anxiety (4, 16). Similarly, high agreeableness, reflecting cooperation and empathy, can foster positive team dynamics and social support, buffering the negative effects of neuroticism (4). Extraversion, associated with sociability and assertiveness, can help players communicate effectively and build confidence, counteracting potential anxiety (4). Research on the direct relationship between neuroticism and athletic performance has yielded mixed results, suggesting that the impact may depend on various contextual factors (4, 16, 19, 24, 30). A study assessing personality traits in team sports using the Five-Factor Model model emphasizes the importance of considering the specific sport, playing position, and individual characteristics when examining the influence of personality on performance (16, 19). However, the null correlations for agreeableness and neuroticism at the global level should be interpreted in light of the more fine-grained findings by playing position and experience. Guards in this sample displayed significantly lower agreeableness than forwards and centers, reflecting the interpersonal demands of assuming ball-dominant, decision-making roles that sometimes require prioritizing rapid, assertive action over maintaining interpersonal harmony (16, 27). At the same time, the absence of performance differences linked to agreeableness suggests that, within elite squads, a range of interpersonal styles can coexist without necessarily producing measurable divergences in statistical efficiency, provided that role expectations and communication structures are clearly defined (16, 29). The experience-related pattern for neuroticism—whereby players with more than ten years of experience reported lower levels of neuroticism than those with five years or less—also points toward a developmental process in which emotionally less stable athletes either acquire effective coping strategies or are progressively filtered out of elite competition (4, 18). This interpretation is consistent with work showing that higher-level and more experienced athletes tend to display greater emotional stability and more adaptive stress-appraisal patterns than their less experienced counterparts, even when exposed to comparable competitive pressures (16, 25). Together, these findings suggest that, in high-level basketball, agreeableness and neuroticism may exert their influence primarily through selection and role allocation processes over the course of a career, rather than through direct, linear associations with match statistics at a single time point. Accordingly, psychological support interventions should focus less on attempting to “normalize” these traits and more on equipping players—especially those in early and mid-career stages—with coping, communication, and conflict-management skills that enable a broad range of personality profiles to function effectively within the team environment (4, 23). A further layer of interpretation concerns the cultural context of the sample, which was drawn from national teams competing in the FIBA Asia Cup qualification system. Cross-cultural research on the Five-Factor Model indicates that mean levels of traits such as extraversion and neuroticism vary systematically across regions and may shape both how personality is expressed and how it relates to performance outcomes (31). Consequently, the pattern of weak or null effects for agreeableness and neuroticism observed here may not generalize directly to athletes from other cultural backgrounds, underlining the importance of replicating these analyses in diverse basketball systems (4, 31). Finally, the overall configuration of results—positive links of openness, conscientiousness, and extraversion with performance, combined with largely indirect roles for agreeableness and neuroticism—is broadly consistent with recent cross-sectional and longitudinal studies in basketball and other sports, which emphasize that personality contributes to performance through multiple, partially overlapping pathways including motivation, training adherence, team processes, and stress management rather than through a single dominant trait (4, 11, 18). Exploring performance of athletic individuals. From an applied perspective, this pattern supports a multidimensional approach to talent identification and player development, in which Five-Factor Model assessments are integrated with technical, tactical, and physical evaluations rather than used as stand-alone selection tools. For example, combining personality profiles with objective performance trajectories can help staff distinguish between athletes who are temporarily underperforming but possess psychological resources conducive to future improvement (e.g., high conscientiousness and openness) and those whose personality configuration may require more intensive psychological skills training or role adjustment to reach their potential (3, 4, 22). In summary, the present findings extend prior work by demonstrating that, within the specific context of professional male basketball in Asia, the Five-Factor Model traits show a differentiated pattern of relationships with both current performance and key structural variables such as playing position and experience level. This underscores the value of incorporating personality assessment into evidence-based coaching practices—such as individualized feedback, role negotiation, and targeted mental skills programs—while also recognizing that personality effects are typically modest in size and should be interpreted alongside broader contextual and performance data (20, 22, 32). The current sample characteristics can explain why agreeableness and neuroticism show no direct links to performance outcomes. The participants were experienced elite athletes competing at the international level, where selection processes and prolonged exposure to high-performance environments may reduce variability in certain personality traits.

Agreeableness may exert its influence primarily through indirect team-level processes, such as cohesion, cooperation, and interpersonal climate, rather than through direct associations with individual performance statistics. The direct effects of neuroticism show limited presence in elite samples because athletes with high emotional instability tend to develop coping strategies or exit high-level competition. The relationship between these traits and direct performance metrics becomes less clear, because the traits may affect team dynamics and psychological functioning in more significant ways. Overall, the results largely support the proposed hypotheses and are consistent with the theoretical framework outlined in the introduction, while also highlighting the modest magnitude of personality performance relationships in elite basketball. From a broader personality perspective, evidence based on the Millon model suggests that performance outcomes are linked to complex motivational, cognitive, and emotional personality configurations rather than to isolated traits alone (8). This supports the present findings, indicating that personality–performance relationships in elite team sports are multifactorial and context-dependent rather than purely trait-specific.

## Conclusion

The study demonstrates that personality traits, particularly openness to experience, conscientiousness, and extraversion, are associated with performance in an incremental manner. It also suggests that disciplined, adaptable, and socially engaged athletes tend to be more efficient and consistent on the court. Although agreeableness and neuroticism were not statistically correlated with performance, their effects on team dynamics and emotional regulation are nonetheless relevant. Hence, the implications for coaches, sport psychologists, and talent scouts highlight the importance of personality assessment. Personality measurement can be incorporated into various aspects of player development and team building. Further studies ought to work upon these findings using longitudinal and multi-source assessments to fully appreciate the dynamic nature of the interplay between personality and performance.

### Limitations

Despite making significant contributions, a number of limitations need to be kept in mind when interpreting and generalizing the present study. First, the sample of this study used only male professional basketball players engaging in the FIBA Asia Cup 2025 qualifications, thus limiting the transferability of the findings to female athletes or athletes of other competitive levels, age groups, or cultural contexts. Future research could obtain more varied samples to investigate possible gender- or culture-related variations in personality–performance relationships. Second, the cross-sectional nature of the study limits the possibility of making causal inferences on personality traits and athletic performance; thus, longitudinal research is recommended to assess how personality dynamics evolve over time and influence performance trajectories. Third, athletic performance was assessed using a customized performance efficiency index based on objective match statistics (points, rebounds, assists, and steals). Although these indicators are widely used and provide an objective representation of on-court performance, the index did not incorporate other potentially relevant metrics, such as personal fouls, minutes played, or plus/minus efficiency ratings. In addition, the external validity of the proposed efficiency index may be limited, as it was developed and applied within the specific context of the FIBA Asia Cup 2025 qualification. Caution is therefore warranted when generalizing the findings to other leagues, competitive levels, or tactical systems. Future studies should seek to validate and refine this performance index by incorporating a broader range of performance indicators across diverse basketball contexts. Last but not least, self-report personality assessments are vulnerable to social desirability and response bias, thus undermining the accuracy of personality profiles, which may inspire multi-source assessment methods (e.g., coach ratings or behavioral observations) in subsequent studies to validate the findings.

## Data Availability

The datasets presented in this study can be found in online repositories. The names of the repository/repositories and accession number(s) can be found in the article/Supplementary Material.
