# Correction: The impact of the five-factor model of personality on the performance of basketball players

**DOI:** 10.3389/fspor.2026.1823696

**Published:** 2026-03-26

**Authors:** Islam Mohammad Abbas

**Affiliations:** Department of Sport Science, Faculty of Sport Science, Arab American University, Jenin, Palestine

**Keywords:** athlete development, basketball performance, big five, personality traits, sports psychology, team sports

The reference for (17) was erroneously written as “Piepiora P, Migasiewicz J, Witkowski K. Personality traits and sports performance: the role of neuroticism in elite athletes. J Sport Psychol. (2024) 35(2):112–29. 10.xxxx/jsp.2024.002”.

It should be “Piepiora, P., Migasiewicz, J., & Napieraj, D. (2019). Personality profile of athletes practising endurance disciplines. Journal of Education, Health and Sport, 9(4), 394–402. Retrieved from https://apcz.umk.pl/JEHS/article/view/6831”.

The reference for (30) was erroneously written as “Piepiora P, Migasiewicz J, Napieraj D. Personality profile of athletes practising endurance disciplines. J Educ Health Sport. (2019) 9(4):394–402. Available online at: https://apcz.umk.pl/JEHS/article/view/6831”.

It should be [Schmitt DP, Allik J, McCrae RR, Benet-Martínez V. The geographic distribution of big five personality traits: patterns and profiles of human self- description across 56 nations. J Cross Cult Psychol. (2007) 38(2):173–212. doi: 10. 1177/0022022106297299].

The reference for (31) was erroneously written as “Schmitt DP, Allik J, McCrae RR, Benet-Martínez V. The geographic distribution of big five personality traits: patterns and profiles of human self-description across 56 nations. J Cross Cult Psychol. (2007) 38(2):173–212. doi: 10. 1177/0022022106297299”.

It should be “Funder DC, Ozer DJ. Evaluating effect size in psychological research: sense and nonsense. Adv Methods Pract Psychol Sci. (2019) 2(2):156–68. doi: 10.1177/2515245919847202”.

The original version of this article has been updated.

