# Supplementary material for: The impact of the five-factor model of personality on the performance of basketball players
Source: Front Sports Act Living. 2026 Mar 4;8:1766462. doi: 10.3389/fspor.2026.1766462 (PMC12996257; doi:10.3389/fspor.2026.1766462)
Supplement: Supplementary file 1 [file Table1.docx]

Supplementary Material

# Supplementary Data

**Table 1: Distribution of Study Sample According to Playing Position & Experience Variable (n=116)**

| **Variable** | **Position Levels** | **Frequency** | **Percentage** |
| --- | --- | --- | --- |
| **Playing Position** | Point Guard | 37 | 31.9% |
|  | Forward | 46 | 39.7% |
|  | Center | 33 | 28.4 |
|  | **Total** | **116** | **100%** |
| **Playing Experience** | 5 ≥ | 35 | 30.2% |
|  | 6 – 10 years | 45 | 38.8% |
|  | > 10 years | 36 | 31% |
|  | **Total** | **116** | **100%** |

**Table 2: Standardized Beta Coefficients and Assigned Relative Weights for Player Performance Variables**

| **Variable** | **Standardized Beta** | **Percentage-Based Relative Weight** |
| --- | --- | --- |
| Points (PTS) | 0.593 | 53.4% |
| Rebounds (REB) | 0.275 | 24.8% |
| Assists (AST) | 0.177 | 15.9% |
| Steals (STL) | 0.066 | 5.9% |

^Note: All independent variables are statistically significant (Sig. < 0.05) with no indication of multicollinearity (VIF < 5), confirming the suitability of the regression model.^

**Table 3: Reliability Coefficients (Cronbach’s Alpha) for the Big Five Personality Traits**

| **Personality Traits** | **Cronbach’s Alpha** | **N of Items** |
| --- | --- | --- |
| openness to experience | 0.814 | 8 |
| conscientiousness | 0.926 | 9 |
| Extraversion | 0.771 | 7 |
| Agreeableness | 0.799 | 8 |
| Neuroticism | 0.735 | 9 |
| **Total Scale Reliability** | 0.909 | 41 |

**Table 4: Results of Pearson’s Correlation Test Between the Big Five Personality Traits and Players Performance**

| **Personality Trait** | **r** | **p-value** | **Statistical significance** |
| --- | --- | --- | --- |
| Openness to Experience | 0.266 | 0.004* | Significant |
| Conscientiousness | 0.228 | 0.001* | Significant |
| Extraversion | 0.187 | 0.045* | Significant |
| Agreeableness | 0.035- | 0.705 | Not Significant |
| Neuroticism | 0.028 | 0.763 | Not Significant |

^*α ≤ 0.05^

**Table 5: Means and standard deviations of the Big Five personality traits among basketball players in Palestine based on playing positions**

| **Playing Position**  **personality traits** | **Point Guard** | | **Forward** | | **Center** | |
| --- | --- | --- | --- | --- | --- | --- |
|  | **Mean** | **Standard Deviation** | **Mean** | **Standard Deviation** | **Mean** | **Standard Deviation** |
| Openness to Experience | 3.40 | 0.835 | 3.51 | 0.806 | 3.53 | 0.751 |
| Conscientiousness | 3.28 | 1.192 | 3.52 | 0.952 | 3.45 | 0.897 |
| Extraversion | 3.31 | 0.704 | 3.57 | 0.830 | 3.48 | 0.912 |
| Agreeableness | 3.37 | 0.610 | 3.72 | 0.388 | 3.80 | 0.587 |
| Neuroticism | 3.32 | 0.556 | 3.49 | 0.645 | 3.29 | 0.652 |

**Table 6: Results of One-Way ANOVA for Differences in Big Five Personality Traits Among Basketball Players by Playing Position**

| **Variables** | **Source of Variation** | **Sum of Squares** | **Degrees of Freedom (df)** | **Mean Square** | **F-value** | **Significance Level (p)** | **Statistical Significance** |
| --- | --- | --- | --- | --- | --- | --- | --- |
| Openness to Experience | Between Groups  Within Groups Total | 0.332  72.508  72.841 | 2  113  115 | 0.166  0.642 | 0.259 | 0.772 | Not Significant |
| Conscientiousness | Between Groups  Within Groups Total | 1.256  117.812  119.068 | 2  113  115 | 0.628  1.043 | 0.602 | 0.549 | Not Significant |
| Extraversion | Between Groups  Within Groups Total | 1.410  75.593  77.004 | 2  113  115 | 0.705  0.669 | 1.054 | 0.352 | Not Significant |
| Agreeableness | Between Groups  Within Groups Total | 3.831  31.241  35.072 | 2  113  115 | 1.915  0.276 | 6.928 | 0.001* | Significant |
| Neuroticism | Between Groups  Within Groups Total | 0.922  43.510  44.431 | 2  113  115 | 0.461  0.385 | 1.197 | 0.306 | Not Significant |

^* Significance Level: (α ≤ 0.05)^

**Table 7: HSD Post Hoc Comparisons (LSD) for Agreeableness Scores by Playing Position**

| **Comparison** | **Mean Difference** | **p-value** | **Statistical Significance** |
| --- | --- | --- | --- |
| Guard vs. Forward | 0.348- | 0.009* | Significant |
| Guard vs. Center | 0.431- | 0.002* | Significant |
| Forward vs. Center | 0.082 | 0.769 | Not Significant |

^* Significance Level: (α ≤ 0.05)^

**Table 8:** **Means And Standard Deviations Of The Big Five Personality Traits Among Basketball Players In Palestine Based On Playing Experience**

| **Playing Experience**  **personality traits** | **5 ≥** | | **6 – 10 years** | | - **>10 years** | |
| --- | --- | --- | --- | --- | --- | --- |
|  | **Mean** | **Standard Deviation** | **Mean** | **Standard Deviation** | **Mean** | **Standard Deviation** |
| Openness to Experience | 3.49 | 0.921 | 3.17 | 0.855 | 3.86 | 0.256 |
| Conscientiousness | 3.40 | 1.023 | 3.10 | 1.272 | 3.85 | 0.165 |
| Extraversion | 3.67 | 0.861 | 3.17 | 0.949 | 3.63 | 0.404 |
| Agreeableness | 3.76 | 0.635 | 3.50 | 0.607 | 3.67 | 0.323 |
| Neuroticism | 3.52 | 0.722 | 3.44 | 0.615 | 3.18 | 0.469 |

**Table 9: Results of One-Way ANOVA for Differences in Big Five Personality Traits Among Basketball Players by Playing Experience**

| **Variables** | **Source of Variation** | **Sum of Squares** | **Degrees of Freedom (df)** | **Mean Square** | **F-value** | **Significance Level (p)** | **Statistical Significance** |
| --- | --- | --- | --- | --- | --- | --- | --- |
| Openness to Experience | Between Groups  Within Groups Total | 9.496  63.344  72.841 | 2  113  115 | 4.748  0.561 | 8.470 | 0.001 | Significant |
| Conscientiousness | Between Groups  Within Groups Total | 11.212  107.856  119.068 | 2  113  115 | 5.606  0.954 | 5.873 | 0.004 | Significant |
| Extraversion | Between Groups  Within Groups Total | 6.367  70.637  77.004 | 2  113  115 | 3.183  0.625 | 5.092 | 0.008 | Significant |
| Agreeableness | Between Groups  Within Groups Total | 1.449  33.623  35.072 | 2  113  115 | 0.724  0.298 | 2.435 | 0.092 | Not Significant |
| Neuroticism | Between Groups  Within Groups Total | 2.302  42.129  44.431 | 2  113  115 | 1.151  0.373 | 3.088 | 0.049 | Significant |

^* Significance Level: (α ≤ 0.05)^

**Table 10: Tukey HSD Post Hoc Comparisons (LSD) for Personality Traits Across Playing Experience**

| **Personality Traits** | **Comparison** | **Mean Difference** | **p-value** | **Statistical Significance** |
| --- | --- | --- | --- | --- |
| Openness to Experience | 5 ≥ vs. **>** 10 years | 0.368- | 0.041* | Significant |
|  | 6 – 10 years vs **>** 10 years | 0.688 | 0.001* | Significant |
| Conscientiousness | 6 – 10 years vs **>** 10 years | 0.748- | 0.001* | Significant |
| Extraversion | 5 ≥ vs. 6 – 10 years | 0.502- | 0.006* | Significant |
|  | 6 – 10 years vs **>** 10 years | 0.456- | 0.011* | Significant |
| Neuroticism | 5 ≥ vs. **>** 10 years | 0.341 | 0.020* | Significant |

^* Significance Level: (α ≤ 0.05)^
